# Lung adenocarcinoma patients have higher risk of SARS-CoV-2 infection

**DOI:** 10.18632/aging.202375

**Published:** 2021-01-10

**Authors:** Long Chen, Yue Liu, Jiamin Wu, Chao Deng, Jianjun Tan, Huawen Liu, Li Zhong

**Affiliations:** 1 Bioengineering Institute of Chongqing University, Chongqing, China; 2 Three Gorges Central Hospital, Wanzhou, Chongqing, China; 3 Chongqing Mix Biotechnology Co., Ltb, Chongqing, China

**Keywords:** lung adenocarcinoma, SARS-CoV-2, ACE2, miR-125b-5p, IL6, COVID-19

## Abstract

Both lung adenocarcinoma and coronavirus disease 2019 would cause pulmonary inflammation. Angiotensin-converting enzyme 2, the functional receptor of SARS-CoV-2, also plays a key role in lung adenocarcinoma. To study the risk of SARS-CoV-2 infection in lung adenocarcinoma patients, mRNA and microRNA profiles were obtained from The Cancer Genome Atlas and Gene Expression Omnibus followed by bioinformatics analysis. A network which regards angiotensin-converting enzyme 2 as the center was structured. In addition, via immunological analysis to explore the essential mechanism of SARS-CoV-2 susceptibility in lung adenocarcinoma. Compared with normal tissue, angiotensin-converting enzyme 2 was increased in lung adenocarcinoma patients. Furthermore, a total of 7 correlated differently expressed mRNAs (ACE2, CXCL9, MMP12, IL6, AZU1, FCN3, HYAL1 and IRAK3) and 5 correlated differently expressed microRNAs (miR-125b-5p, miR-9-5p, miR-130b-5p, miR-381-3p and miR-421) were screened. Interestingly, the most frequent toll-like receptor signaling pathway was enriched by mRNA (interlukin 6) and miRNA (miR-125b-5p) sets simultaneously. In conclusion, it was assumed that miR-125b-5p-ACE2-IL6 axis could alter the risk of SARS-CoV-2 infection in lung adenocarcinoma patients.

## INTRODUCTION

In December 2019, there was an outbreak of coronavirus pneumonia (COVID-19) caused by SARS-CoV-2 in Wuhan, Hubei province in China. SARS-CoV-2 could encode spike (S) protein like SARS-CoV. In addition, a same cell entry receptor and manner were used to mediate infection. Unlike other type I fusion proteins, the S protein of coronaviruses is not cleaved in the virus-producing cell [1, 2]. However, in processed coronaviruses, two domains (S1 and S2) with different functions can be defined [3]. The S1 domain induces receptor association, whereas the S2 domain likely undergoes structural rearrangements that mediate membrane fusion. A discrete receptor-binding domain (RBD) of S protein has been defined at residues 318-510 of the S1 domain. This RBD could bind receptor with higher affinity than full S1 domain [4, 5].

Angiotensin-converting enzyme 2 (ACE2), a terminal carboxypeptidase, catalyzes the conversion of angiotensin II (Ang II) to angiotensin 1-7 (Ang 1-7). Ang II, the major effector molecule of the conventional renin-angiotensin system, is implicated in the pathogenesis of cardiovascular disorders like hypertension, atherosclerosis, and myocardial infarction. Whereas ACE2 and its product Ang 1-7 are thought to prevent the detrimental effects of angiotensin II [6]. In respiratory organs, ACE2 is the receptor for SARS-CoV [7, 8]. As a type I transmembrane protein, ACE2 is comprised of a short cytoplasmic domain, a transmembrane domain, and a large ectodomain [9]. The ACE2 ectodomain, includes the first α-helix and N-terminus proximal residues of β-sheet, could bind the SARS-CoV S glycoprotein with high affinity [10]. Furthermore, once SARS-CoV binds to ACE2, the abundance on the cell surface, the mRNA expression and enzymatic activity of ACE2 are significantly reduced. ACE2 has also been shown to attenuate inflammation and acute lung injury caused by SARS-CoV infection mainly through inhibition of angiotensin II/NF-κB signaling [11–15]. The ACE2 dysfunction is implicated in SARS pathogenesis. ACE2 is predominantly localized on the apical surface of well-differentiated airway epithelia, especially ciliated cells, and also been identified with the pathology of various inflammatory lung diseases like lung cancer [16–18]. Furthermore, the expression of ACE2 might be influenced by many factors like organ, gender, age, development, lifestyle (cigarette smoking, diet) and some diseases (chronic obstructive pulmonary disease, hypertension, coronary heart disease, chronic kidney disease and diabetes) [19]. Abundant clinical data has verified that the ACE2 level would alter infection risk of SARS-cov-2. For example, COVID-19 patients with comorbidities were more likely to progress to critically ill patients compared with those without comorbidities [20].

Lung adenocarcinoma (LUAD), the most common primary lung cancer which falls under the umbrella of non-small cell lung cancer (NSCLC), has a strong association with smoking. LUAD usually evolves from the mucosal glands and represents about 40% of lung cancers. LUAD usually occurs in the lung periphery, and in many cases, may be found in scars or areas of chronic inflammation [21, 22]. The tumor microenvironment of LUAD may also regulate ACE2 expression level which alters the risk of SARS-CoV-2 infection. Furthermore, there are lots of factors that could cause both ACE2 level alteration and fluctuation of immune-regulation. Thus, through bioinformatics analysis and network structuring, the potential mechanism of COVID-19 in LUAD patient might be explored.

## RESULTS

### The expression level of ACE2 in LUAD

**Figure 1:**
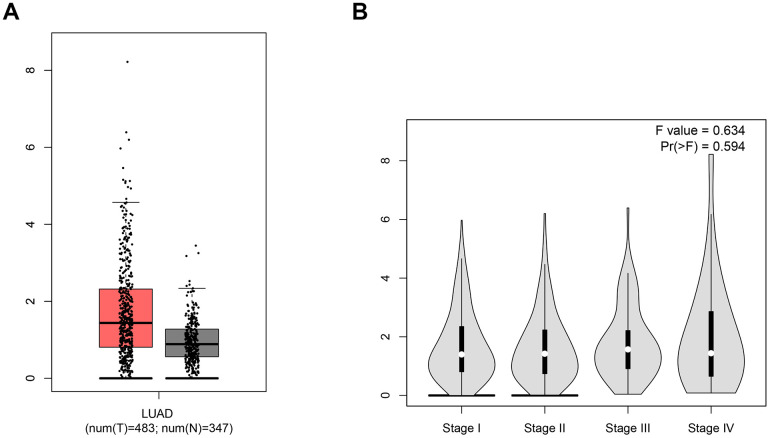
**The expression level of ACE2.** (**A**) The box-plot showed the comparison of ACE2 mRNA level in normal tissues vs. LUAD tissues. The red samples represent tissues from LUAD patients and the gray ones represent tissues from healthy persons. (**B**) The violin plot showed the expression level of ACE2 in different stages of LUAD. The parameters were listed in the upper right.

Ang 1-7, the product of ACE2, was confirmed to inhibit the proliferation of human lung cancer cells through interaction with the Mas receptor (MasR) [23]. Ang1-7 not only reduces the size of human lung tumor xenografts *in vivo* but also markedly decreases their vessel density [24–26]. Thus, the expression level of ACE2 in LUAD would influence the cancer progression. To compare the ACE2 level between LUAD and normal tissues, sequencing data of a total of 830 samples (347 normal tissues and 483 LUAD tissues) were obtained from the TCGA database (Figure 1). In LUAD patients, a higher expression level of ACE2 was detected while there was no significant difference among various stages of LUAD. Due to the function of combining with S glycoprotein, higher ACE2 level might lead to increased risk of SARS-CoV-2 infection.

### The regulatory network revolves around ACE2

**Figure 2:**
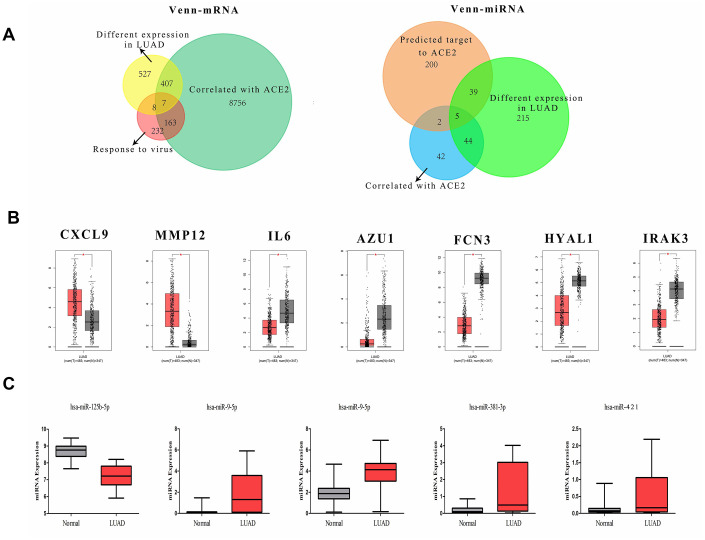
**The definition of DECGs and DECMs.** (**A**) Two Venn-plots showed the sifting process of DECGs (left) and DECMs (right). The yellow circle represents the differentially expressed mRNA in LUAD. The green circle represents the genes correlated with ACE2 in LUAD. The carnation circle represents the gene set involved in virus defense. The orange circle represents the miRNAs which could bind to 3’-UTR of ACE2. The aqua circle represents the differentially expressed miRNAs in LUAD. The blue circle represents the miRNAs correlated with ACE2 in LUAD. (**B**, **C**) The box-plots showed the comparison of DECGs or DECMs level in normal tissues vs. LUAD tissues. The red samples represent tissues from LUAD patients and the gray ones represent tissues from healthy persons.

**Figure 3:**
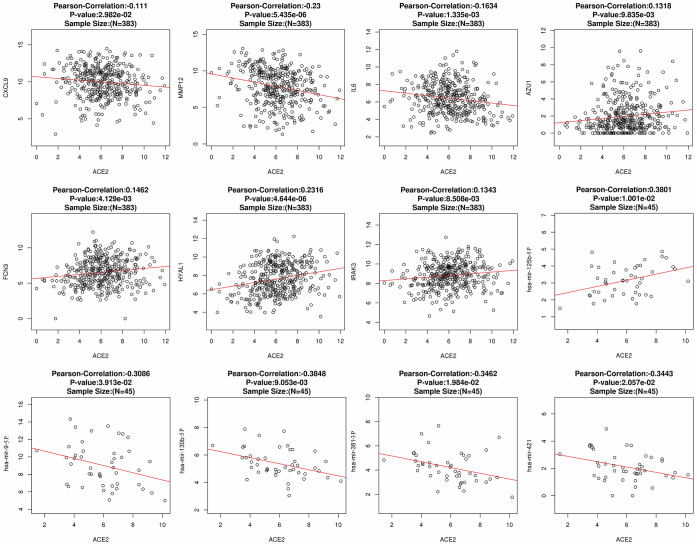
**The correlation between ACE2 and DECGs or DECMs.** Each hollow circle represents a single sample. The red line represents the correlation. A total of 383 and 45 samples were obtained to calculate the correlation between ACE2 and DECGs or DECMs, respectively.

The fluctuation of gene expression often involves complicated regulatory networks. To study the potential mechanism of ACE2 dysregulation in LUAD, the relevant molecular around ACE2 were identified. After screening and comparison, a total of 7 differentially expressed correlative genes (DECGs) and 5 differentially expressed correlative miRNAs (DECMs) were detected respectively. In addition, the DECGs (CXCL9, MMP12, IL6, AZU1, FCN3, HYAL1, IRAK3) were involved in multi defense virus processes and the DECMs (miR-125b-5p, miR-9-5p, miR-130b-5p, miR-381-3p, miR-421) were predicted to target the 3’ untranslated regions (UTR) of ACE2 (Figure 2 and Supplementary Figure 1). Among them, CXCL9, MMP12, miR-9-5p, miR-130b-5p, miR-381-3p and miR-421 were increased while IL6, AZU1, FCN3, HYAL1, IRAK3 and miR-125b-5p were reduced in LUAD patients. Meanwhile, AZU1, FCN3, HYAL1, IRAK3 and miR-125b-5p were positively correlated with ACE2 while CXCL9, MMP12, IL6, miR-9-5p, miR-130b-5p, miR-381-3p, and miR-421 were negatively correlated (Figure 3). Notably, only miR-125b-5p has an opposite expression trend with ACE2. Thus, miR-125b-5p was assumed to be the single upstream predicted inhibitor of ACE2. In addition, among the 7 DECGs, IL6 owns the highest mutation frequency (5%) in LUAD patients might indicate that IL6 could mediate carcinogenesis (Supplementary Figure 2).

**Figure 4:**
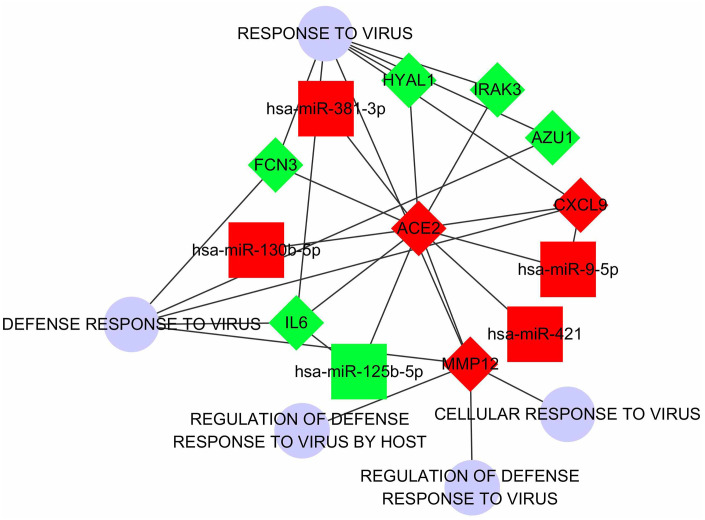
**The regulatory network which regards ACE2 as the center.** The squares represent DECMs. The rhombus represents DECGs. The blue circles represent biological processes correlated with virus defense. The red nodes represent the up-regulation of DECGs or DECMs. The green nodes represent down-regulation of DECGs or DECMs. The edges between every 2 nodes represent subordination or interactive relationship.

To understand the involved pathways and functions of the DECGs and DECMs in LUAD, enrichment analysis was performed (Supplementary Figure 3). A total of 41 pathways and 129 GO functions were enriched based on DECGs and DECMs. Interestingly, both 2 DECGs (CXCL9, IL6) and 4 DECMs (miR-125b-5p, miR-9-5p, miR-130b-5p, and miR-421) were enriched in the toll-like receptor signaling pathway (Supplementary Figure 4). In this pathway, CXCL9 takes part in chemotactic effects of T cells while IL6 participates in proinflammatory effects. Then according to the relationships between DECGs, DECMs and virus-associated processes, an interaction network was constructed (Figure 4). Notably, not only ACE2 but IL6 were predicted as the targets of miR-125b-5p. Similarly, both ACE2 and CXCL9 were regarded as the predicted target of miR-9-5p. However, due to the expression trend of CXCL9 and miR-9-5p were not opposite with ACE2, it was suggested that both CXCL9 and miR-9-5p may not play a decisive role in ACE2 expression.

### Immunological function of ACE2 and IL6

**Figure 5:**
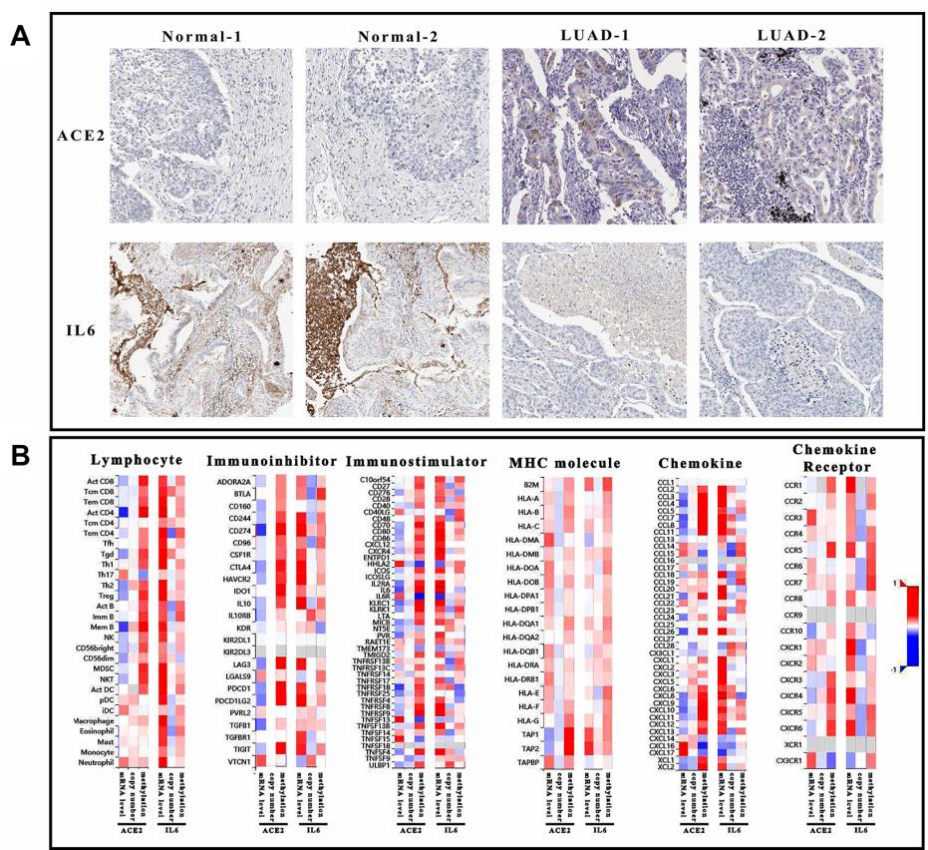
**Immunological analysis of ACE2 and IL6.** (**A**) The pathological sections of tumor tissue and normal tissue which was stained by ACE2 and IL6 antibody. Each group has 2 duplicate samples. (**B**) The heatmap showed the correlation of 6 immunological factors (lymphocyte, immune-inhibitor, immune-stimulator, MHC molecule, chemokine, and chemokine receptor) vs. mRNA expression, copy number and methylation of ACE2 and IL6.

In conclusion, the reduced miR-125b-5p might be the primary inhibitor of ACE2 in LUAD. Once ACE2 was dysregulated, IL6 in toll-like receptor pathway might activate the immune system as a downstream effector. No matter the defense of SARS-CoV-2 or pneumonia in LUAD, the altered immunoreaction was the primary cause. Thus, the ability of ACE2 and IL6 to regulate immune system was evaluated based on the correlation between gene characters (mRNA expression, copy number and methylation) and immune elements (lymphocyte, immune-inhibitor, immune-stimulator, MHC molecule, chemokine, and chemokine receptor) (Figure 5). Interestingly, CXCL9 could mediate immunoreaction as a chemokine which also influenced by ACE2 and IL6 (expression level and methylation). Therefore, it was suggested that even CXCL9 might not participate in regulating ACE2 level, it would like to mediate immune-regulation of ACE2 and IL6.

## DISCUSSION

Once infected with SARS-CoV-2, the pathogenic T cells were activated rapidly followed by immune response and virus eliminating. However, the fatal cause of viral pneumonia was the uncontrolled inflammation. In addition, previous study has demonstrated that COVID-19 might be more likely to cause an endothelial damage in the vessel system. ACE2, not only as the functional receptor of coronavirus, also emerged as a potent negative regulator of the RAS which maintaining homeostasis of blood pressure and inflammatory responses [27]. Altered activation of the RAS is often attributed to the pathogenesis of many diseases such as hypertension, myocardial infarction and inflammatory lung disease [28, 29]. Compared with normal COVID-19 patients, whether the vessel system of LUAD has higher ACE2 level is unclear. In murine acute respiratory distress syndrome models, it was confirmed that the lack of ACE2 expression in the lung resulted in attenuated vascular permeability, enhanced lung edema, neutrophil infiltration, and further deteriorated lung function [30]. Thus, though higher ACE2 level would increase the risk of SARS-CoV-2 infection, the normal level of ACE2 would also improve prognosis of patients with acute lung injury.

The RAS contained ACE2 participate in maintaining homeostasis of inflammatory responses. However, once SARS-CoV-2 binds to ACE2, the abundance on the cell surface, mRNA expression and the enzymatic activity of ACE2 are significantly reduced which lead to the inflammatory storm. Coincidentally, IL6 was the main factor which could also induce an inflammatory storm. Once infected with SARS-CoV-2, the activated pathogenic T cells could produce granulocyte-macrophage colony-stimulating factors (GM-CSF) and IL6. The GM-CSF could further activate CD14+CD16+ inflammatory monocytes. In addition, more cytokines include IL6 were produced via positive feedback loop. A large concentration of immune cells and tissue fluid in the lungs can block gas exchange between alveoli and capillaries, leading to acute respiratory distress syndrome. Once a cytokine storm forms, the immune system would kill lots of normal cells in the lung while eliminating the virus, severely damaging the lung's ventilation function. In a word, similar to ACE2, IL6 play key role in defense of SARS-CoV-2 with normal expression level. Once deregulated, IL6 will become a lethal factor for patients.

Conventionally, the LUAD patients have lower immunity than healthy persons. The increased ACE2 level in the lung might lead to susceptibility to infection. Nevertheless, the pathology of COVID-19 is complicated, except miRNA introduced in present study, transcription factor, lncRNA and ceRNA may participate in mechanism of COVID. The result of our study may provide a reference for further research.

## MATERIALS AND METHODS

### Sequencing datasets

Dataset GSE74190 was acquired from GEO Dataset. All experiments were approved by the local ethics committee. Expression profiles of 723 human miRNAs were investigated in selected cancerous cells and normal cells populations derived from 36 LUAD and 44 adjacent normal tissues. ALL cells derived from 82 snap-frozen surgical specimens. The sequencing platform is Agilent-019118 Human miRNA Microarray V1 G4470A [miRBase release 9.1 miRNA ID version].

### MiRNA target gene prediction and analysis of affected signaling pathway

The potential target genes of differentially expressed miRNAs in LUAD were acquired from the widely used online databases TargetScanHuman 7.2 [31] and DIANA-microT [32]. To reduce false positives, the predicted target genes which appeared at both databases were accepted. Following this, the list of predicted target genes of individual miRNAs was imported to DIANA-mirPath, a miRNA pathway analysis web server. Canonical pathways significantly affected by individual differentially expressed miRNAs were made a contrast and then we obtained the common pathways of which elements were also as the targets of deregulation miRNAs. All these canonical pathways were identified from the Kyoto Encyclopaedia of Genes and Genomes (KEGG) databases.

### Microarray data and enrichment analysis

Total RNA from tissues was isolated using Trizol extractions (Invitrogen). The RNA quantity was assessed by NanoDrop®ND-1000 spectrophotometer and RNA 6000 NanoChips with the Agilent 2100 Bioanalyzer (Agilent, Palo Alto, CA, USA). 100 ng of total RNA was amplified using the Ambion® WT Expression Kit (4411973, Life Technologies). Small RNA libraries were prepared using 1μg of total RNA according to the TruSeq Small RNA Sample Preparation Guide (Illumina, San Diego, CA, USA). To generate count data, the raw sequences were compared to human mature miRNA sequences (from miRBase version 17) and non-coding RNA sequences (Rfam version 10) by MEGABLAST.

Background deletion, quantile normalization, and probe assembly were performed. Different expression mRNAs between normal vs. LUAD samples were detected by the R package DESeq [33]. P-values were adjusted for multiple comparisons via using the Benjamini-Hochberg procedure [34]. MiRNA with an adjusted p-value of < 0.05 and logFC ≥ 2.0 was considered as differentially expressed. Gene and miRNA enrichment analyses were performed with DAVID version 6.7 and DIANA- mirPATH v.3 respectively. The enriched biological GO and pathway terms were identified [35]. The interaction network was drawn by Cytoscape. Some other databases used are listed in Table 1.

**Table 1:** List of databases.

**Database ID**	**URL**
GEO Dataset	https://www.ncbi.nlm.nih.gov/gds/?term=
TCGA	https://www.cancer.gov/
cBioportal of cancer genomics	https://www.cbioportal.org/
DSA	http://cancer.digitalslidearchive.net/
The Human Protein Atlas	https://www.proteinatlas.org/
Linked Omics	http://www.linkedomics.org/
Targetscan	http://www.targetscan.org/vert_72/
OncomiR	http://www.oncomir.org/oncomir/index.html
DAVID	https://david.ncifcrf.gov/
DIANA-mirPATH v.3	http://diana.imis.athena-innovation.gr/DianaTools/index.php
DIANA-microT	http://diana.imis.athena-innovation.gr/DianaTools/index.php?r=microT_CDS/index
TIMER	https://cistrome.shinyapps.io/timer/
STRING	https://string-db.org/
GEPIA	http://gepia.cancer-pku.cn/index.html
Pathview	https://pathview.uncc.edu/
TISIDB	http://cis.hku.hk/TISIDB/index.php

### Statistical analyses

Results are presented as mean values ± standard error of the mean (SEM). Unless mentioned otherwise, the statistical comparison between groups was performed by using t-test, a maximum of three comparisons were performed per panel, and robustness of statistical significance was verified after correction for multiple testing. Probability was considered to be significant at p < 0.05.

### Data availability statement

Publicly available datasets were analyzed in this study. All data was available.

## Supplementary Material

Supplementary Figures

## References

[1] Xiao X, Chakraborti S, Dimitrov AS, Gramatikoff K, Dimitrov DS. The SARS-CoV S glycoprotein: expression and functional characterization. Biochem Biophys Res Commun. 2003; 312:1159–64. 10.1016/j.bbrc.2003.11.054 14651994 PMC7111010

[2] Moore MJ, Dorfman T, Li W, Wong SK, Li Y, Kuhn JH, Coderre J, Vasilieva N, Han Z, Greenough TC, Farzan M, Choe H. Retroviruses pseudotyped with the severe acute respiratory syndrome coronavirus spike protein efficiently infect cells expressing angiotensin-converting enzyme 2. J Virol. 2004; 78:10628–35. 10.1128/JVI.78.19.10628-10635.2004 15367630 PMC516384

[3] Gallagher TM, Buchmeier MJ. Coronavirus spike proteins in viral entry and pathogenesis. Virology. 2001; 279:371–74. 10.1006/viro.2000.0757 11162792 PMC7133764

[4] Wong SK, Li W, Moore MJ, Choe H, Farzan M. A 193-amino acid fragment of the SARS coronavirus S protein efficiently binds angiotensin-converting enzyme 2. J Biol Chem. 2004; 279:3197–201. 10.1074/jbc.C300520200 14670965 PMC7982343

[5] Babcock GJ, Esshaki DJ, Thomas WD Jr, Ambrosino DM. Amino acids 270 to 510 of the severe acute respiratory syndrome coronavirus spike protein are required for interaction with receptor. J Virol. 2004; 78:4552–60. 10.1128/jvi.78.9.4552-4560.2004 15078936 PMC387703

[6] Jiang F, Yang J, Zhang Y, Dong M, Wang S, Zhang Q, Liu FF, Zhang K, Zhang C. Angiotensin-converting enzyme 2 and angiotensin 1-7: novel therapeutic targets. Nat Rev Cardiol. 2014; 11:413–26. 10.1038/nrcardio.2014.59 24776703 PMC7097196

[7] Hamming I, Timens W, Bulthuis ML, Lely AT, Navis G, van Goor H. Tissue distribution of ACE2 protein, the functional receptor for SARS coronavirus. A first step in understanding SARS pathogenesis. J Pathol. 2004; 203:631–37. 10.1002/path.1570 15141377 PMC7167720

[8] Kuba K, Imai Y, Rao S, Gao H, Guo F, Guan B, Huan Y, Yang P, Zhang Y, Deng W, Bao L, Zhang B, Liu G, et al. A crucial role of angiotensin converting enzyme 2 (ACE2) in SARS coronavirus-induced lung injury. Nat Med. 2005; 11:875–79. 10.1038/nm1267 16007097 PMC7095783

[9] Tipnis SR, Hooper NM, Hyde R, Karran E, Christie G, Turner AJ. A human homolog of angiotensin-converting enzyme. Cloning and functional expression as a captopril-insensitive carboxypeptidase. J Biol Chem. 2000; 275:33238–43. 10.1074/jbc.M002615200 10924499

[10] Li W, Zhang C, Sui J, Kuhn JH, Moore MJ, Luo S, Wong SK, Huang IC, Xu K, Vasilieva N, Murakami A, He Y, Marasco WA, et al. Receptor and viral determinants of SARS-coronavirus adaptation to human ACE2. EMBO J. 2005; 24:1634–43. 10.1038/sj.emboj.7600640 15791205 PMC1142572

[11] Jia HP, Look DC, Tan P, Shi L, Hickey M, Gakhar L, Chappell MC, Wohlford-Lenane C, McCray PB Jr. Ectodomain shedding of angiotensin converting enzyme 2 in human airway epithelia. Am J Physiol Lung Cell Mol Physiol. 2009; 297:L84–96. 10.1152/ajplung.00071.2009 19411314 PMC2711803

[12] Liu Z, Huang XR, Chen HY, Penninger JM, Lan HY. Loss of angiotensin-converting enzyme 2 enhances TGF-β/smad-mediated renal fibrosis and NF-κB-driven renal inflammation in a mouse model of obstructive nephropathy. Lab Invest. 2012; 92:650–61. 10.1038/labinvest.2012.2 22330342

[13] Meng Y, Yu CH, Li W, Li T, Luo W, Huang S, Wu PS, Cai SX, Li X. Angiotensin-converting enzyme 2/angiotensin-(1-7)/Mas axis protects against lung fibrosis by inhibiting the MAPK/NF-κB pathway. Am J Respir Cell Mol Biol. 2014; 50:723–36. 10.1165/rcmb.2012-0451OC 24168260

[14] Zou Z, Yan Y, Shu Y, Gao R, Sun Y, Li X, Ju X, Liang Z, Liu Q, Zhao Y, Guo F, Bai T, Han Z, et al. Angiotensin-converting enzyme 2 protects from lethal avian influenza a H5N1 infections. Nat Commun. 2014; 5:3594. 10.1038/ncomms4594 24800825 PMC7091848

[15] Tao L, Qiu Y, Fu X, Lin R, Lei C, Wang J, Lei B. Angiotensin-converting enzyme 2 activator diminazene aceturate prevents lipopolysaccharide-induced inflammation by inhibiting MAPK and NF-κB pathways in human retinal pigment epithelium. J Neuroinflammation. 2016; 13:35. 10.1186/s12974-016-0489-7 26862037 PMC4748536

[16] Yamaguchi M, Hirai S, Sumi T, Tanaka Y, Tada M, Nishii Y, Hasegawa T, Uchida H, Yamada G, Watanabe A, Takahashi H, Sakuma Y. Angiotensin-converting enzyme 2 is a potential therapeutic target for EGFR-mutant lung adenocarcinoma. Biochem Biophys Res Commun. 2017; 487:613–18. 10.1016/j.bbrc.2017.04.102 28433633 PMC7092918

[17] Qian YR, Guo Y, Wan HY, Fan L, Feng Y, Ni L, Xiang Y, Li QY. Angiotensin-converting enzyme 2 attenuates the metastasis of non-small cell lung cancer through inhibition of epithelial-mesenchymal transition. Oncol Rep. 2013; 29:2408–14. 10.3892/or.2013.2370 23545945

[18] Jia H. Pulmonary angiotensin-converting enzyme 2 (ACE2) and inflammatory lung disease. Shock. 2016; 46:239–48. 10.1097/SHK.0000000000000633 27082314

[19] Li Y, Zhou W, Yang L, You R. Physiological and pathological regulation of ACE2, the SARS-CoV-2 receptor. Pharmacol Res. 2020; 157:104833. 10.1016/j.phrs.2020.104833 32302706 PMC7194807

[20] Guan WJ, Ni ZY, Hu Y, Liang WH, Ou CQ, He JX, Liu L, Shan H, Lei CL, Hui DS, Du B, Li LJ, Zeng G, et al, and China Medical Treatment Expert Group for Covid-19. Clinical characteristics of coronavirus disease 2019 in China. N Engl J Med. 2020; 382:1708–20. 10.1056/NEJMoa2002032 32109013 PMC7092819

[21] Li C, Lu H. Adenosquamous carcinoma of the lung. Onco Targets Ther. 2018; 11:4829–35. 10.2147/OTT.S164574 30147334 PMC6098426

[22] Myers DJ, Wallen JM. Cancer, Lung Adenocarcinoma, in StatPearls. 2020, StatPearls Publishing StatPearls Publishing LLC: Treasure Island (FL).

[23] Raizada MK, Ferreira AJ. ACE2: a new target for cardiovascular disease therapeutics. J Cardiovasc Pharmacol. 2007; 50:112–19. 10.1097/FJC.0b013e3180986219 17703127

[24] Bernardi S, Zennaro C, Palmisano S, Velkoska E, Sabato N, Toffoli B, Giacomel G, Buri L, Zanconati F, Bellini G, Burrell LM, De Manzini N, Fabris B. Characterization and significance of ACE2 and Mas receptor in human colon adenocarcinoma. J Renin Angiotensin Aldosterone Syst. 2012; 13:202–09. 10.1177/1470320311426023 22048948

[25] Gallagher PE, Tallant EA. Inhibition of human lung cancer cell growth by angiotensin-(1-7). Carcinogenesis. 2004; 25:2045–52. 10.1093/carcin/bgh236 15284177

[26] Menon J, Soto-Pantoja DR, Callahan MF, Cline JM, Ferrario CM, Tallant EA, Gallagher PE. Angiotensin-(1-7) inhibits growth of human lung adenocarcinoma xenografts in nude mice through a reduction in cyclooxygenase-2. Cancer Res. 2007; 67:2809–15. 10.1158/0008-5472.CAN-06-3614 17363603

[27] Balakumar P, Jagadeesh G. A century old renin-angiotensin system still grows with endless possibilities: AT1 receptor signaling cascades in cardiovascular physiopathology. Cell Signal. 2014; 26:2147–60. 10.1016/j.cellsig.2014.06.011 25007996

[28] del Castillo Rueda A, Guerrero Sanz JE, Escalante Cobo JL, Grau Carmona T, de Portugal Alvarez J. [Serum and pulmonary angiotensin converting enzyme as a marker of acute lung injury in an experimental model of adult respiratory distress syndrome]. An Med Interna. 1999; 16:229–35. 10389307

[29] Gonzalez NC, Allen J, Schmidt EJ, Casillan AJ, Orth T, Wood JG. Role of the renin-angiotensin system in the systemic microvascular inflammation of alveolar hypoxia. Am J Physiol Heart Circ Physiol. 2007; 292:H2285–94. 10.1152/ajpheart.00981.2006 17208999

[30] Imai Y, Kuba K, Rao S, Huan Y, Guo F, Guan B, Yang P, Sarao R, Wada T, Leong-Poi H, Crackower MA, Fukamizu A, Hui CC, et al. Angiotensin-converting enzyme 2 protects from severe acute lung failure. Nature. 2005; 436:112–16. 10.1038/nature03712 16001071 PMC7094998

[31] Lewis BP, Shih IH, Jones-Rhoades MW, Bartel DP, Burge CB. Prediction of mammalian microRNA targets. Cell. 2003; 115:787–98. 10.1016/s0092-8674(03)01018-3 14697198

[32] Kiriakidou M, Nelson PT, Kouranov A, Fitziev P, Bouyioukos C, Mourelatos Z, Hatzigeorgiou A. A combined computational-experimental approach predicts human microRNA targets. Genes Dev. 2004; 18:1165–78. 10.1101/gad.1184704 15131085 PMC415641

[33] Anders S, Huber W. Differential expression analysis for sequence count data. Genome Biol. 2010; 11:R106. 10.1186/gb-2010-11-10-r106 20979621 PMC3218662

[34] Klipper-Aurbach Y, Wasserman M, Braunspiegel-Weintrob N, Borstein D, Peleg S, Assa S, Karp M, Benjamini Y, Hochberg Y, Laron Z. Mathematical formulae for the prediction of the residual beta cell function during the first two years of disease in children and adolescents with insulin-dependent diabetes mellitus. Med Hypotheses. 1995; 45:486–90. 10.1016/0306-9877(95)90228-7 8748093

[35] Huang da W, Sherman BT, Lempicki RA. Systematic and integrative analysis of large gene lists using DAVID bioinformatics resources. Nat Protoc. 2009; 4:44–57. 10.1038/nprot.2008.211 19131956

