# Supplementary material for: Lung adenocarcinoma patients have higher risk of SARS-CoV-2 infection
Source: Aging (Albany NY). 2021 Jan 10;13(2):1620–32. doi: 10.18632/aging.202375 (PMC7880402; doi:10.18632/aging.202375)
Supplement: Supplementary Figures [file aging-13-202375-s001.pdf]

SUPPLEMENTARY FIGURES

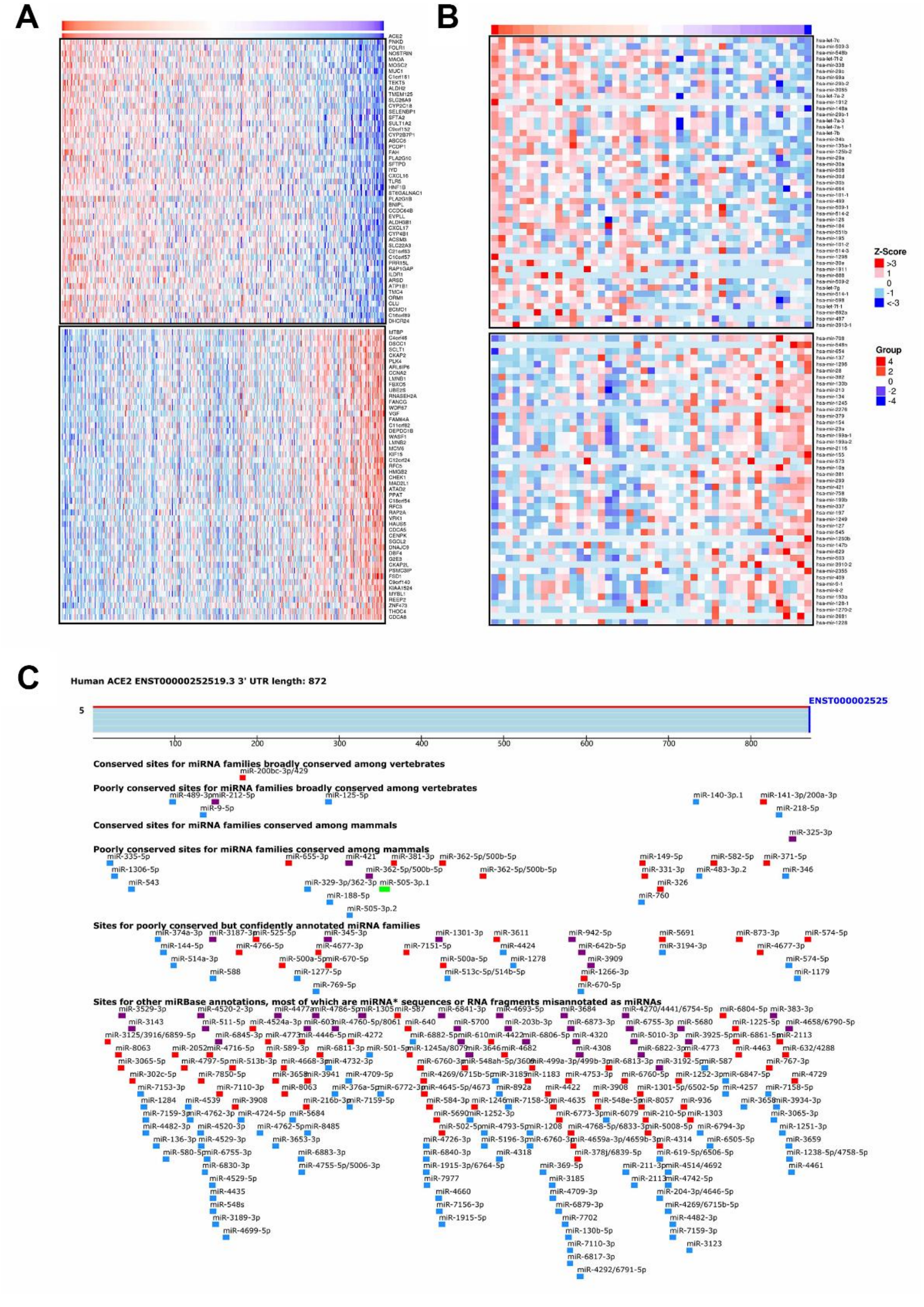

Supplementary Figure 1. The correlated mRNAs (**A**) and miRNAs (**B**) of ACE2 in LUAD. Due to the number of correlated genes is too large, only top 50 mRNAs were list. The red point represents positive correlation while the blue ones represents negative correlation. (**C**) The sketch map of predicted target sites of miRNAs in ACE2 3'-UTR. Different colors represent the site with various probability of preferential conservation.

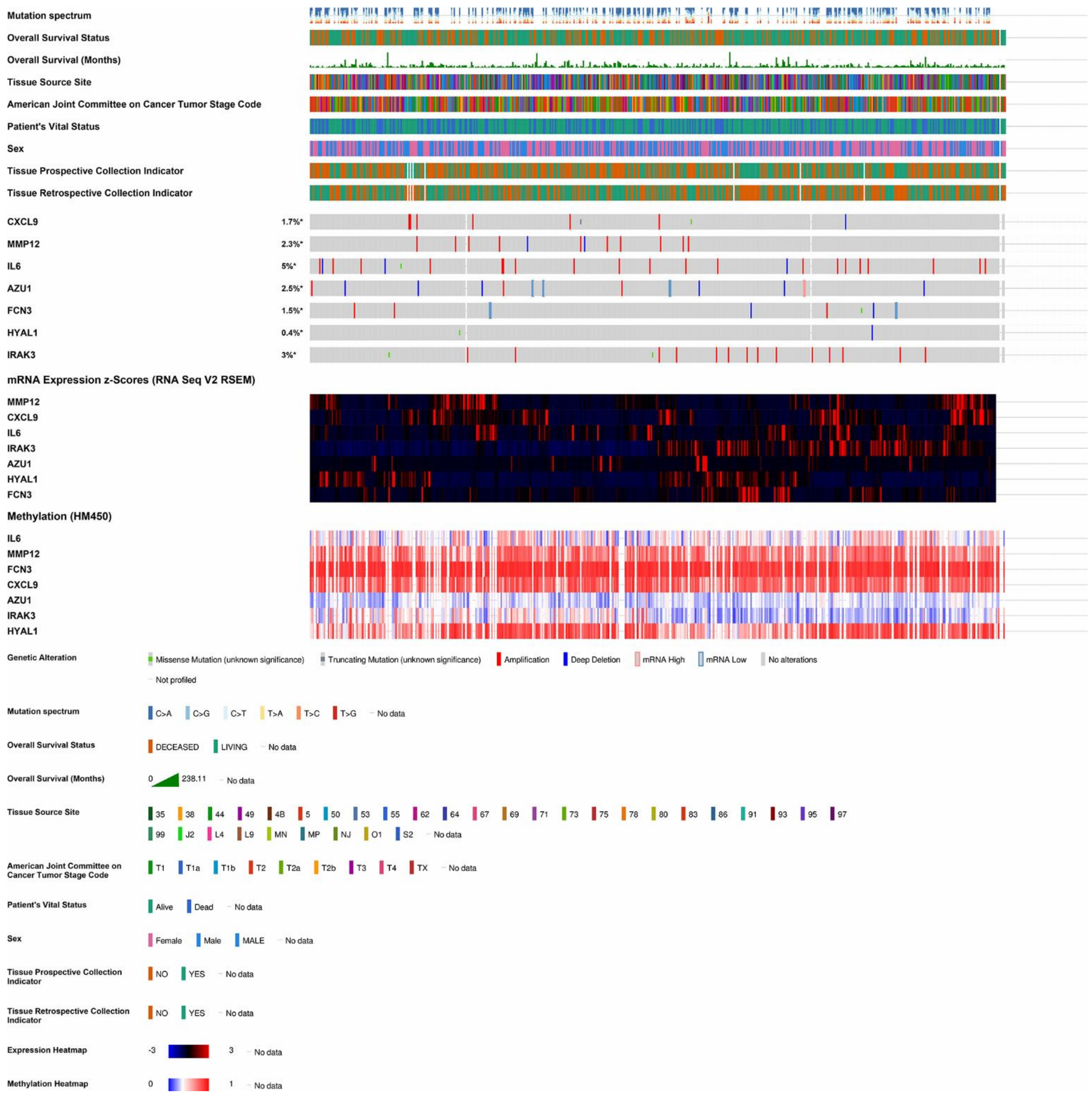

Supplementary Figure 2. Integrated plot of clinical data and 7 mutation of DECGs in 586 LUAD samples. From top to bottom panels indicate: mutation spectrum, overall survival status, overall survival (months), tissue source site, American Joint Committee on Cancer tumor stage code, patients' vital status, sex, tissue prospective collection indicator, tissue retrospective collection indicator, mutation symbol of DECGs, heatmap of DECGs expression and methylation. The key to the color-coding is at the bottom.

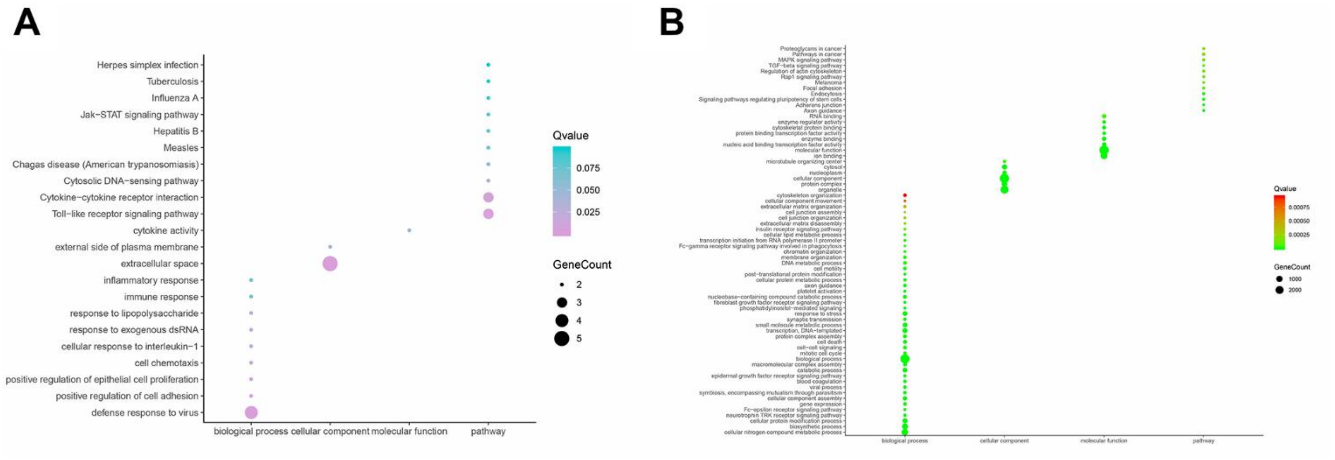

Supplementary Figure 3. The GO enrichment and KEGG pathway analysis of down-regulated (**A**) and up-regulated (**B**) genes, respectively. The legends are on the right.

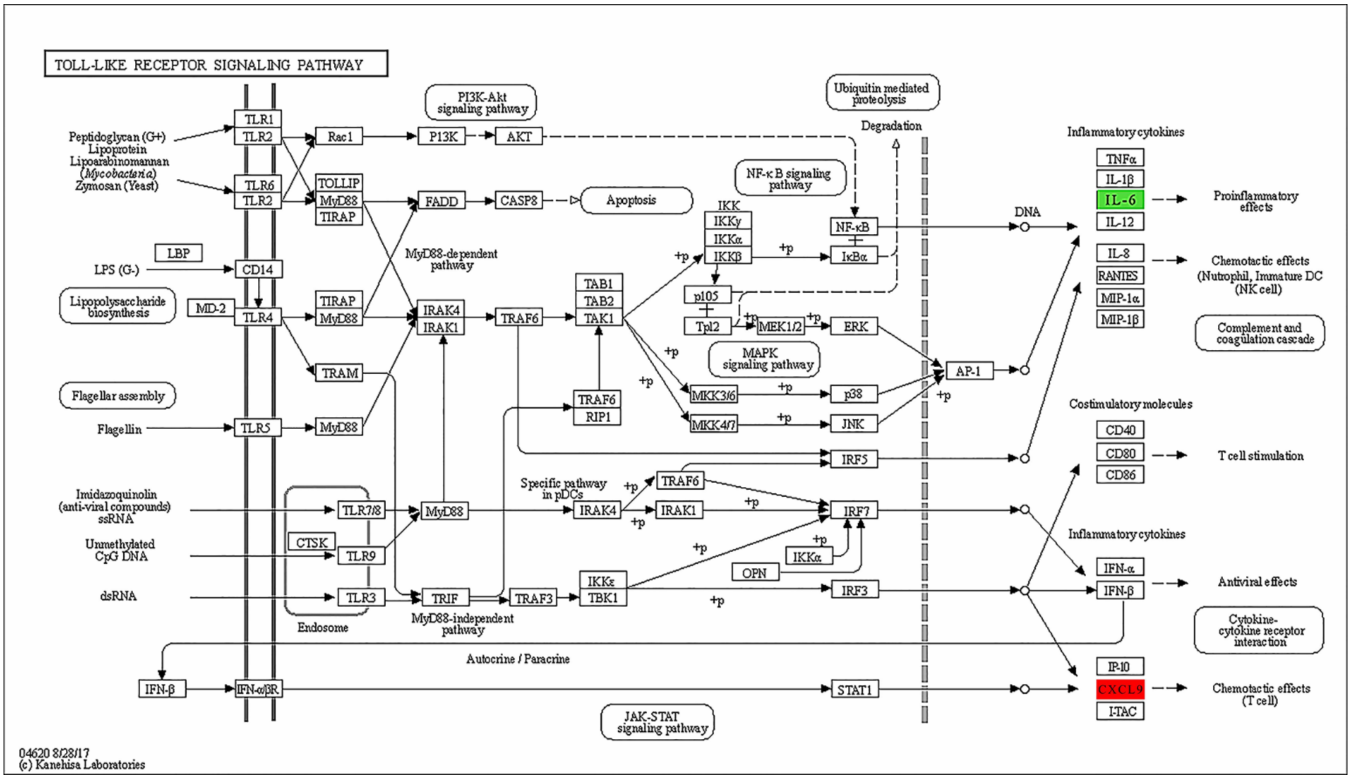

Supplementary Figure 4. The sketch map of toll-like receptor signaling pathway. The red box and green box represent up-regulated and down-regulated gene, respectively.
